# Sphk1 and Sphk2 Differentially Regulate Erythropoietin Synthesis in Mouse Renal Interstitial Fibroblast-like Cells

**DOI:** 10.3390/ijms23115882

**Published:** 2022-05-24

**Authors:** Redona Hafizi, Faik Imeri, Bisera Stepanovska Tanturovska, Roxana Manaila, Stephanie Schwalm, Sandra Trautmann, Roland H. Wenger, Josef Pfeilschifter, Andrea Huwiler

**Affiliations:** 1 Institute of Pharmacology, Inselspital, INO-F, University of Bern, CH-3010 Bern, Switzerland; redona.hafizi@pki.unibe.ch (R.H.); faik.imeri@dbmr.unibe.ch (F.I.); bisera.stepanovska@pki.unibe.ch (B.S.T.); roxana.manaila@pki.unibe.ch (R.M.); 2 Institute of General Pharmacology and Toxicology, University Hospital Frankfurt am Main, Goethe-University, Theodor-Stern Kai 7, D-60590 Frankfurt am Main, Germany; s.schwalm@med.uni-frankfurt.de (S.S.); pfeilschifter@em.uni-frankfurt.de (J.P.); 3 Institute of Clinical Pharmacology, University Hospital Frankfurt am Main, Goethe-University, Theodor-Stern Kai 7, D-60590 Frankfurt am Main, Germany; trautmann@med.uni-frankfurt.de; 4 Institute of Physiology, University of Zürich, CH-8057 Zürich, Switzerland; roland.wenger@access.uzh.ch

**Keywords:** Sphk2, Sphk1, sphingosine, sphingosine 1-phosphate, HIF-2α, erythropoietin, renal fibroblasts, anemia

## Abstract

Erythropoietin (Epo) is a crucial hormone regulating red blood cell number and consequently the hematocrit. Epo is mainly produced in the kidney by interstitial fibroblast-like cells. Previously, we have shown that in cultures of the immortalized mouse renal fibroblast-like cell line FAIK F3-5, sphingosine 1-phosphate (S1P), by activating S1P_1_ and S1P_3_ receptors, can stabilize hypoxia-inducible factor (HIF)-2α and upregulate Epo mRNA and protein synthesis. In this study, we have addressed the role of intracellular iS1P derived from sphingosine kinases (Sphk) 1 and 2 on Epo synthesis in F3-5 cells and in mouse primary cultures of renal fibroblasts. We show that stable knockdown of Sphk2 in F3-5 cells increases HIF-2α protein and Epo mRNA and protein levels, while Sphk1 knockdown leads to a reduction of hypoxia-stimulated HIF-2α and Epo protein. A similar effect was obtained using primary cultures of renal fibroblasts isolated from wildtype mice, *Sphk1*−/−, or *Sphk2*−/− mice. Furthermore, selective Sphk2 inhibitors mimicked the effect of genetic *Sphk2* depletion and also upregulated HIF-2α and Epo protein levels. The combined blockade of Sphk1 and Sphk2, using *Sphk2*−/− renal fibroblasts treated with the Sphk1 inhibitor PF543, resulted in reduced HIF-2α and Epo compared to the untreated *Sphk2*−/− cells. Exogenous sphingosine (Sph) enhanced HIF-2α and Epo, and this was abolished by the combined treatment with the selective S1P_1_ and S1P_3_ antagonists NIBR-0213 and TY52156, suggesting that Sph was taken up by cells and converted to iS1P and exported to then act in an autocrine manner through S1P_1_ and S1P_3_. The upregulation of HIF-2α and Epo synthesis by Sphk2 knockdown was confirmed in the human hepatoma cell line Hep3B, which is well-established to upregulate Epo production under hypoxia. In summary, these data show that sphingolipids have diverse effects on Epo synthesis. While accumulation of intracellular Sph reduces Epo synthesis, iS1P will be exported to act through S1P_1+3_ to enhance Epo synthesis. Furthermore, these data suggest that selective inhibition of Sphk2 is an attractive new option to enhance Epo synthesis and thereby to reduce anemia development in chronic kidney disease.

## 1. Introduction

Sphingosine 1-phosphate (S1P) is a key regulator of many physiological and pathophysiological processes in almost every organ [1,2,3]. It is synthesized intracellularly from sphingosine (Sph) by two sphingosine kinases, Sphk1 and Sphk2 [4,5], and is degraded either reversibly by unspecific lipid phosphate phosphatases and specific S1P phosphatases [6], or irreversibly by the enzyme S1P lyase [7].

S1P primarily acts as a high-affinity ligand to a family of cell surface S1P receptors (S1PR), which includes the five subtypes S1P_1–5_ [8]. These receptors belong to the superfamily of G protein-coupled receptors (GPCR) and couple to various G proteins, including heterotrimeric G_i/0_, G_q_, but also small G proteins, which activate signaling pathways such as the mitogen-activated-protein kinase (MAPK) cascade, the PI3K/Akt cascade, and protein kinase C (PKC) [8,9]. Furthermore, S1P may also act as an intracellular second messenger to regulate Ca^2+^ homeostasis and antagonize apoptotic effects. Both sites of S1P action result in distinct activation of different signaling cascades, cytoskeletal rearrangement, endothelial cell permeability, and cell migration [1,2,4,5].

In the kidney, S1P signaling contributes to inflammatory and fibrotic processes during chronic kidney disease (CKD) [3,10,11]. Interestingly, S1P, generated by either Sphk1 or Sphk2, has distinct roles in CKD. By using genetic knockout mice of the two kinases in various models of renal fibrosis, it was shown that loss of *Sphk2* is protective and reduces disease parameters, such as in the unilateral ureteral obstruction (UUO) [12,13,14] and diabetic nephropathy models [15], while inhibition of Sphk1 is still not conclusive because in one study it aggravated fibrosis [16], but in another study reduced fibrosis [17]. The reason why S1P appears to elicit opposite outcomes of a cellular response is still not fully understood, but it is very likely due to differential subcellular sites of S1P generation and action. Notably, knockout of *Sphk1* or *Sphk2* also has opposite effects on blood plasma S1P levels: depletion of *Sphk1* reduces plasma S1P [18] while depletion of *Sphk2* increases plasma S1P [19]. It is well possible that depleting the one enzyme redirects the substrate sphingosine into the other enzyme’s reaction path [20,21,22,23].

In various studies, it was demonstrated that extracellular S1P (eS1P) and iS1P exert antagonistic effects on certain cell responses. eS1P has been linked to a pro-fibrotic effect by cross-activating the TGFβ/Smad signaling cascade and upregulating pro-fibrotic genes like connective tissue growth factor CTGF [24,25,26] and other fibrotic factors such as α-smooth muscle actin (α-SMA) and collagen [27,28]. In contrast, iS1P was suggested to have antifibrotic effects. Ren et al. showed that in human podocytes, the application of a “caged S1P” analog, which upon illumination liberates iS1P, reduced CTGF expression in a similar way as overexpression of Sphk1, while the knockdown of Sphk1 upregulated TGFβ-stimulated CTGF [29].

In renal tubulointerstitial fibrosis, the key cells participating in extracellular matrix (ECM) production are the interstitial fibroblasts, although other cell types, such as peritubular pericytes, fibrocytes, or tubular epithelial cells, may also contribute to increased ECM production [30,31]. In previous studies, it was shown that the anti-fibrotic effect observed after *Sphk2* depletion in the mouse UUO model in vivo, was also seen in interstitial fibroblasts isolated from *Sphk2−/−* mice in vitro. These cells produced less fibrotic factors upon TGFβ stimulation [12]. A sub-fraction of renal interstitial fibroblasts is also considered the main site of erythropoietin (Epo) synthesis [32,33,34].

Epo is the key growth factor regulating red blood cell homeostasis in mammals [35,36]. Epo is regulated by hypoxia-inducible factor (HIF)-2α-dependent transcriptional activation [37]. In adults, renal Epo synthesis by interstitial fibroblast-like cells is the primary source of circulating Epo, contributing up to 90% of total Epo [38]. Renal Epo-producing cells (REPCs) possess cellular plasticity and undergo a phenotypic transition to myofibroblasts upon inflammatory stimuli which governs fibrotic processes [39]. Activated NFκB- and Smad-signaling pathways repress Epo production and stimulate REPC transformation to myofibroblasts in a UUO mouse model. Alongside the progression of kidney fibrosis during late stages of CKD, patients develop Epo-dependent renal anemia [40,41,42]. Treatment with recombinant Epo may result in cardiovascular complications, hospitalization, and increased mortality rate [43]. However, the detailed mechanisms in interstitial fibroblasts that lead to reduced Epo synthesis and renal fibrosis remain unclear.

In a recent study, we showed that in the immortalized mouse REPC-derived cell line FAIK F3-5, extracellular S1P (eS1P) stimulated Epo synthesis by enhancing HIF-2α stabilization [44]. However, the role of iS1P on Epo production is still not addressed. Thus, in this study, we aimed to investigate the role of iS1P on Epo synthesis in mouse renal fibroblast-like REPCs. We found that Sphk1 and Sphk2 exert opposite functions in regulating Epo synthesis. While inhibition or depletion of Sphk1 reduced Epo synthesis, inhibition or depletion of Sphk2 rather enhanced Epo synthesis. Mechanistically, we suggest that inhibition of Sphk2 leads to Sph accumulation that is directed into the Sphk1 reaction to produce more S1P for export and action through S1P_1+3_. When completely blocking all conversion of Sph to S1P, by blocking Sphk1 plus Sphk2 together, exogenous Sph was no longer able to stimulate Epo synthesis.

These data suggest that there are different subcellular pools of S1P and Sph, that may have differential effects on Epo synthesis. Most importantly, our data suggest that Sphk2 is an attractive novel pharmacological target to upregulate Epo synthesis, which may be useful to prevent anemia in CKD patients.

## 2. Results

In our previous study, we have shown that in the mouse REPC-derived cell line FAIK F3-5, eS1P, via S1P_1_ and S1P_3_ receptor activation, causes HIF-2α stabilization and thereby increases Epo mRNA and protein expression. Now, we investigated, which impact the S1P-generating enzymes Sphk1 and Sphk2 may have on Epo production in F3-5 cells.

**Figure 1:**
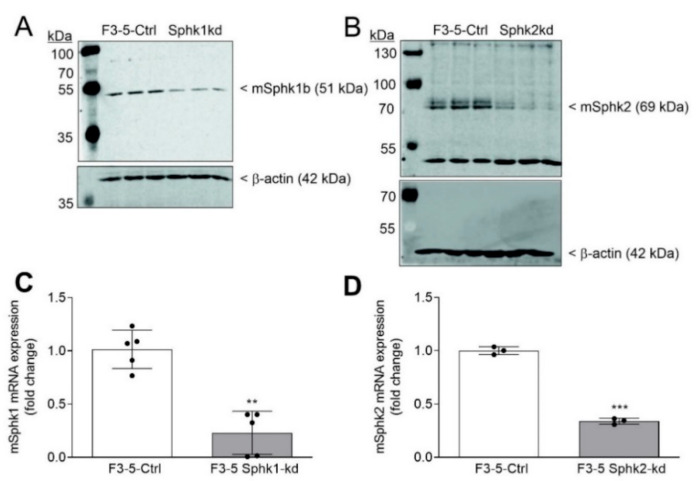
Stable mSphk1- and mSphk2-knockdown in F3-5 cells. F3-5 cells stably transduced with either an empty lentiviral vector (Ctrl) or a lentiviral vector transcribing shRNA targeting mSphK1 (Sphk1-kd), or mSphk2 (Sphk2-kd) mRNA, were incubated for 16 h in serum-free DMEM. Protein (**A**,**B**) and RNA (**C**,**D**) were extracted. Proteins were analyzed by immunoblotting using antibodies against mSphk1 (**A**, upper panel), mSphk2 (**B**, upper panel), or β-actin (**A**,**B**, lower panels). RNA was taken for quantitative PCR analysis using primers for mSphk1 (**C**) and mSphk2 (**D**), and 18S RNA for normalization. Results are expressed as fold change and are means ± S.D. (*n* = 5 or 3). ** *p* < 0.01, *** *p* < 0.001 considered statistically significant when compared to the control cells.

To this end, we transduced F3-5 cells with lentiviral particles transcribing shRNAs targeting mSphk1 and mSphk2 mRNAs, or a control construct (Ctrl), to generate stable knockdown cell lines for these enzymes. For each enzyme, 4–5 different shRNAs were used and the knockdown efficiency was tested on protein level. The best knockdown was obtained with sh5 for mSphk1 (80% reduction) (Figure 1A) and a combination of sh1 + sh3 + sh4 for mSphk2 (75% reduction) (Figure 1B). These two cell lines were taken for further studies. The knockdown cell lines were also tested for cross-regulation of the remaining kinase. We found that the Sphk2-kd cells reacted with an upregulation of Sphk1, an effect that was already previously reported in other studies [20,21,22] (Figure S1). Sphk2 protein was unaltered in Sphk1-kd. To see the impact of this cross-regulation on the cellular S1P and Sph levels, these lipids and the dihydro species were quantified in cells by mass spectrometry. As expected, Sphk1-kd cells had reduced S1P levels, but unchanged Sph levels, while Sphk2-kd cells showed unchanged S1P but enhanced Sph levels (Figure S2).

**Figure 2:**
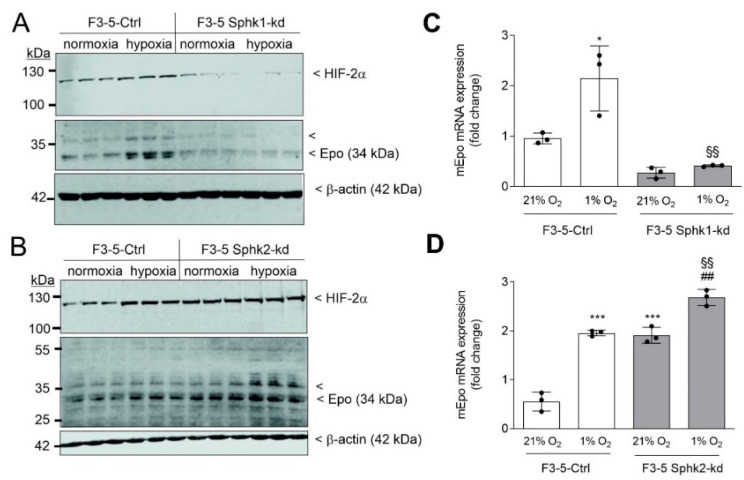
Sphk1- and Sphk2-knockdown in F3-5 cells affects hypoxia-induced HIF-2α protein and Epo mRNA and protein levels. Control F3-5 cells (Ctrl), or cells stably downregulating mSphk1 (**A**,**C**, Sphk1-kd) and mSphk2 (**B**,**D**, Sphk2-kd) were exposed for 6 h to normoxia (21% oxygen) or hypoxia (1% oxygen). Proteins (**A**,**B**) were analyzed by immunoblotting using antibodies against HIF-2α (top panels), Epo (middle panels), or β-actin (bottom panels). Corresponding bands were evaluated by using Image Studio Lite software, normalized to β-actin, and are presented in Figure S3. mRNA (**C**,**D**) was quantified by qPCR analysis using primers for mouse Epo, and L28 RNA for normalization. Results are expressed as fold change and are means ± S.D. (*n* = 3). * *p* < 0.05, *** *p* < 0.001 were considered statistically significant when compared to the normoxic Ctrl cells; ^##^ *p*< 0.01 compared to the respective normoxic kd cells; ^§§^ *p* < 0.01 compared to the respective hypoxic Ctrl cells.

Knockdown (kd) cells were exposed for 6 h to normoxia (21% O_2_) or hypoxia (1% O_2_). In control (Ctrl) F3-5 cells, hypoxia typically upregulated Epo protein levels when compared to normoxia controls. This hypoxia effect was abolished in Sphk1-kd cells (Figure 2A), while it was upregulated in Sphk2-kd cells (Figure 2B). Notably, a band at 35–40 kDa is also detected by the anti-Epo antibody, which likely represents a higher glycosylated intracellular form of Epo [45]. HIF-2α, the key transcription factor regulating Epo transcription in hypoxia, was regulated in a similar manner, i.e., hypoxia-induced HIF-2α protein levels were reduced in Sphk1-kd, but upregulated in Sphk2-kd. Notably, HIF-2α was already increased in normoxic Sphk2-kd cells.

**Figure 3:**
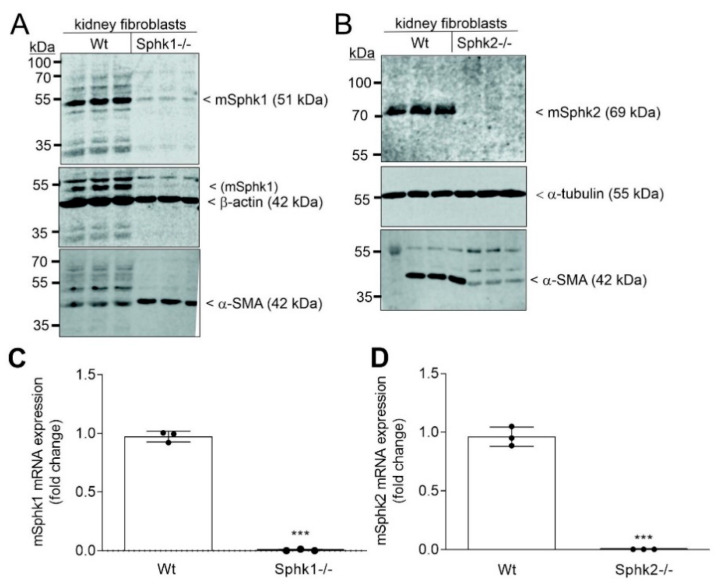
Loss of Sphk expression in primary cultures of kidney fibroblasts isolated from *Sphk1−/−* and *Sphk2−/−* mice. Cells were isolated from C57BL/6 (Wt), *Sphk1−/−*, and *Sphk2−/−* mice. Confluent cells in culture were incubated for 16 h in serum free DMEM and analyzed by immunoblotting using antibodies against mSphk1 (**A**, top panel), mSphk2 (**B**, top panel), β-actin (**A**, middle panel), α-tubulin (**B**, middle panel), and α-smooth muscle actin (α-SMA) (**A**,**B**, bottom panel). mRNA was taken for qPCR analysis using primers for mSphk1 (**C**) and mSphk2 (**D**), and 18S rRNA for normalization. Results are expressed as fold change and are means ± S.D. (*n* = 3). *** *p* < 0.001 considered statistically significant when compared to Wt cells.

To confirm the results obtained with immortalized F3-5 cells, primary kidney fibroblasts were isolated from either wildtype (Wt) control C57BL/6, *Sphk1−/−*, or *Sphk2−/−* mice. The respective enzymes were completely lost in the respective cultures of primary cells (Figure 3A,B). All three cell isolations were positive for α-SMA demonstrating that the cells were of mesenchymal origin. Notably, compared to Wt fibroblasts, α-SMA protein was decreased in *Sphk2−/−* fibroblasts, but enhanced in *Sphk1−/−* fibroblasts, which confirms previous reports that *Sphk2* deficiency downregulates the fibrotic phenotype of the cells [12,13,14], while *Sphk1* deficiency enhances the fibrotic phenotype [16,29]. Knockout cells were also tested for cross-regulation of the remaining kinase (Figure S4). *Sphk2−/−* cells again showed an upregulation of Sphk1 (Figure S4) similar as seen in F3-5 cells, while *Sphk1−/−* expressed unaltered Sphk2 protein. Lipid quantifications revealed that only *Sphk1−/−* cells had reduced S1P and dihydro-S1P levels (Figure S5). Remarkably, *Sphk2−/−* had strongly increased Sph and dihydro-Sph levels, while S1P and dihydro-S1P were slightly increased, possibly by redirecting Sph to the Sphk1 reaction (Figure S5).

**Figure 4:**
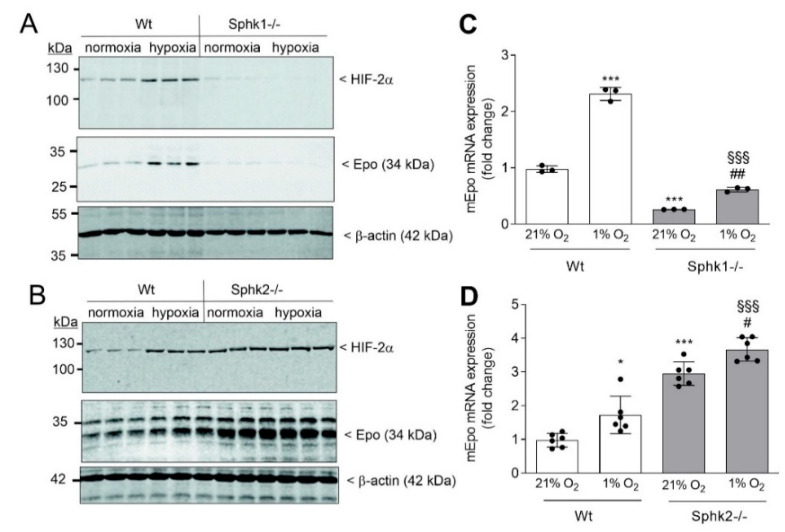
Sphk1 and Sphk2 deficiency affects hypoxia-induced HIF-2α and Epo protein and mRNA expression in primary cultures of fibroblasts. *Sphk1−/−* (**A**), or *Sphk2−/−* (**B**) mouse kidney fibroblasts were incubated for 16 h in serum-free DMEM prior to exposure for 6 h to normoxia (21% oxygen) or hypoxia (1% oxygen). Protein was analyzed by immunoblotting using antibodies against HIF-2α (top panels), Epo (middle panels), or β-actin (bottom panels). Corresponding bands were evaluated by using Image Studio Lite software (version 3.1, LI-COR Biosciences, Lincoln, NE, USA), normalized to β-actin, and are presented in Figure S6. mRNA (**C**,**D**) was quantified by qPCR using primers for mouse Epo, or L28 mRNA for normalization. Results are expressed as fold change and are means ± S.D. (*n* = 3 or 6); * *p* < 0.05, *** *p* < 0.001 were considered statistically significant when compared to the normoxic Ctrl cells; ^#^ *p* < 0.05, ^##^ *p* < 0.01 compared to the *Sphk1−/−* or *Sphk2−/−* normoxic cells; ^§§§^ *p* < 0.001 compared to the respective hypoxic Ctrl cells.

These cells were then exposed to hypoxia or normoxia, and HIF-2α and Epo protein levels were determined. Again, in *Sphk1−/−*, hypoxic induction of HIF-2α was lost (Figure 4A), while it was upregulated in *Sphk2−/−* cells (Figure 4B). A similar regulation was seen for Epo protein (Figure 4A,B, middle panels) and for Epo mRNA expression (Figure 4C,D).

**Figure 5:**
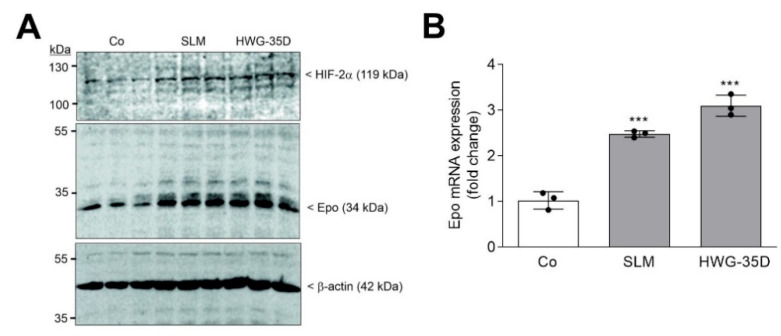
The selective Sphk2 inhibitors SLM6031434 and HWG-35D affect HIF-2α and Epo in primary mouse fibroblasts. Confluent wildtype primary fibroblasts were incubated for 16 h in serum-free DMEM prior to stimulation for 6 h with either vehicle (Co) of 3 µM of SLM6031434 (SLM), or HWG-35D. (**A**) Protein was analyzed by immunoblotting using antibodies against HIF-2α (top panel), Epo (middle panel), or β-actin (bottom panel). Corresponding bands were evaluated by Image Studio Lite software (version 3.1, LI-COR Biosciences, Lincoln, NE, USA), normalized to β-actin and are presented in Figure S7. (**B**) mRNA was analyzed by qPCR using primers for mEpo, and mL28 mRNA for normalization. ΔΔCt values were calculated and results show the fold change compared to the untreated control and are means ± S.D. (*n* = 3), *** *p* < 0.001 were considered statistically significant when compared to the control samples.

To investigate whether the Epo-enhancing effect by loss of *Sphk2* can be mimicked by a Sphk2 inhibitor, we treated wildtype primary fibroblasts for 24 h with the two highly selective Sphk2 inhibitors SLM6031434 and HWG-35D. Both inhibitors were previously shown to block Sphk2 activity in vitro [46,47], as well as in vivo [48]. Both inhibitors, at a concentration of 3 µM, enhanced HIF-2α protein and upregulated Epo protein (Figure 5A) and mRNA expression (Figure 5B).

**Figure 6:**
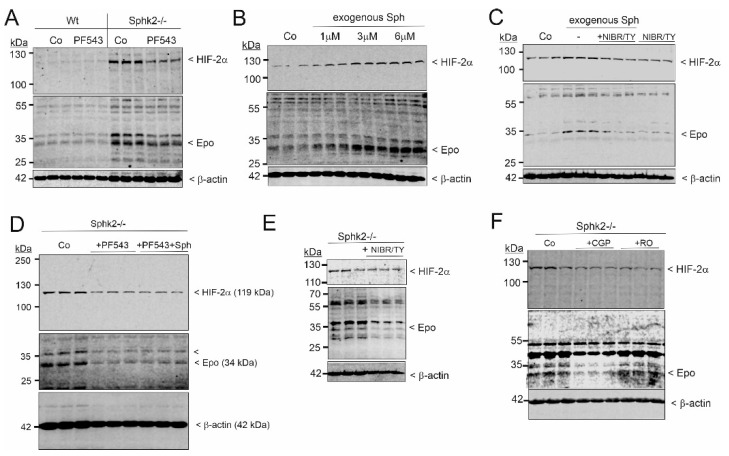
Exogenous sphingosine and the Sphk1 inhibitor PF543 affect HIF-2α and Epo protein levels in primary cultures of mouse fibroblasts. (**A**): Wildtype (Wt) and *Sphk2−/−* renal fibroblasts were incubated for 16 h with serum-free DMEM prior to stimulation for 24 h with 1µM PF543. (**B**) Wt fibroblasts were treated for 24 h with increasing concentrations of exogenous sphingosine. (**C**) Wt fibroblasts were treated for 24 h with 3 µM sphingosine in the absence (−) or presence of 10 µM each of NIBR-0213 plus TY52156. (**D**) *Sphk2−/−* renal fibroblasts were pretreated for 4 h with 1 µM of PF543 and then treated for 24 h with 3 µM sphingosine. (**E**) *Sphk2−/−* cells were treated for 24 h with 10 µM NIBR-0213 and TY52156. (**F**) *Sphk2−/−* cells were treated for 24 h with 100 nM CGP41251 or 1 µM RO-318220. Protein was analyzed by immunoblotting using antibodies against HIF-2α, Epo, or β-actin. Corresponding bands were evaluated by using Image Studio Lite software (version 3.1, LI-COR Biosciences, Lincoln, NE, USA), normalized to β-actin, and are presented in Figure S9.

Since loss or inhibition of Sphk2 leads to increased cellular levels of sphingosine, which could feed into the Sphk1 system, we also tested the impact of a combined blockade of Sphk1 and Sphk2 by using *Sphk2−/−* cells treated for 24 h with a 1 µM concentration of the selective Sphk1 inhibitor PF543. Under these conditions, Epo synthesis was reduced (Figure 6A), suggesting that Sph, which accumulates strongly when both Sphks are blocked (Figure S8), may inhibit HIF-2α stabilization and Epo upregulation. However, treatment of Wt cells with exogenous Sph did not reduce, but rather enhanced HIF-2α and Epo in a concentration-dependent manner (Figure 6B). This was blocked by the combination of the selective S1P_1_ antagonist NIBR-0213 [49] and the selective S1P_3_ antagonist TY52156 [50] (Figure 6C), suggesting that exogenous Sph feeds directly into the Sphk1 pathway and thus generates iS1P for immediate export and S1PR activation. Under Sphk1 and Sphk2 double blockade, exogenous Sph was no longer able to increase HIF-2α and Epo protein levels (Figure 6D). Furthermore, in *Sphk2−/−* cells, HIF-2α and Epo were reduced in the presence of the S1P_1_ and S1P_3_ antagonists NIBR-0213 and TY52156 together (Figure 6E), but also by the two PKC inhibitors CGP41521 and Ro-318220 (Figure 6F), thus further suggesting that the effect of *Sphk2−/−* on Epo is due to secreted S1P and autocrine action through its receptors and PKC signaling.

Similar to hypoxia, the iron chelating drug deferoxamine [51] also increased HIF-2α and Epo protein levels and this effect was blocked by the Sphk1 inhibitor PF543 and the combined antagonism of S1P_1_ and S1P_3_ (Figure S10).

**Figure 7:**
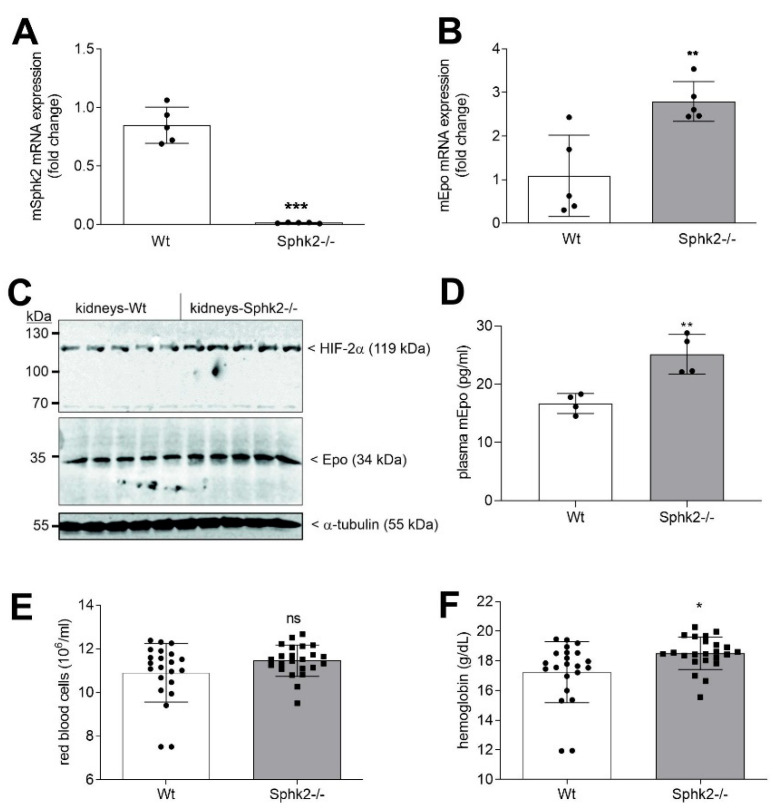
Epo mRNA and protein levels in kidneys from Wt and *Sphk2−/−* mice under normoxic conditions. Kidneys from C57BL/6 (Wt) mice and *Sphk2−/−* mice were taken for RNA (**A**,**B**) or protein (**C**) extraction. mRNA was analyzed by qPCR using primers for mSphk2 (**A**) and mEpo (**B**), and mL28 mRNA for normalization. ΔΔCt values were calculated and results show the fold change compared to the Wt controls and are means ± S.D. (*n* = 5), ** *p* < 0.01, *** *p* < 0.001 considered statistically significant when compared to the Wt control samples. (**C**) Protein was analyzed by immunoblotting using antibodies against HIF-2α (top panel), Epo (middle panel), and α-tubulin (bottom panel). Corresponding bands were evaluated by Image Studio Lite software (version 3.1, LI-COR Biosciences, Lincoln, NE, USA), normalized to α-tubulin, and are presented in Figure S11. (**D**) Blood was collected from Wt or *Sphk2−/−* mice and plasma Epo was quantified by ELISA. Results are depicted in pg/mL and are means ± S.D. (*n* = 4), ** *p* < 0.01 was considered statistically significant when compared to the Wt samples. (**E**,**F**) Blood from Wt and *Sphk2−/−* mice was analyzed for the number of red blood cells (**E**) and hemoglobin (**F**) as described in Section 4. Results are means ± S.D. (*n* = 22 for Wt and 23 for *Sphk2−/−*). * *p* < 0.05 compared to the Wt mice.

To examine whether the upregulating effect of *Sphk2* deficiency on Epo synthesis is also seen in renal fibroblasts in vivo, kidneys from *Sphk2−/−* and control C57BL/6 mice were analyzed for Epo protein and mRNA expression. Figure 7A shows a complete loss of Sphk2 mRNA confirming the *Sphk2* gene knockout, while Epo mRNA (Figure 7B), Epo protein (Figure 7C), as well as HIF-2α protein (Figure 7C) were significantly increased in *Sphk2−/−* kidneys compared to control kidneys. Moreover, Epo in mouse plasma was also enhanced in *Sphk2−/−* (Figure 7D). To see whether this has a consequence on the number of red blood cells in *Sphk2−/−* mice, we re-analyzed the red blood cells and the hemoglobin level from our previous studies on *Sphk2−/−* where we had determined lymphocyte numbers [52,53,54]. Retrospectively, data from 22 Wt mice and 23 *Sphk2−/−* mice were pooled and show increased hemoglobin and a trend to increased red blood cells in *Sphk2−/−* (Figure 7E,F). 

**Figure 8:**
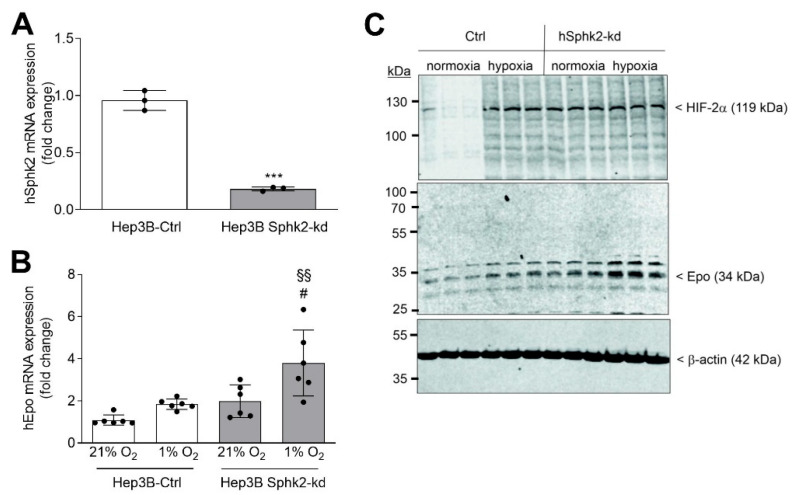
hSphk2 knockdown affects hypoxia-induced HIF-2α and Epo protein expression in the human hepatoma cell line Hep3B. Hep3B cells were transduced with a control shRNA construct (Ctrl) or with a hSphk2 shRNA construct (hSphk2-kd), and stably selected cells were either exposed for 6 h to normoxia (21% oxygen) or hypoxia (1% oxygen). (**A**,**B**) mRNA was analyzed by qPCR using primers of hSphk2 (**A**) and hEpo (**B**), and 18S (for hSphk2) and hL28 (for Epo) mRNA for normalization. ΔΔCt values were calculated and normalized to 18S or L28, respectively. Results are expressed as fold change compared to normoxic control cells and are means +/− S.D. (*n* = 6), *** *p* < 0.001 was considered statistically significant when compared to the normoxic control; ^#^ *p* < 0.05 compared to the normoxic hSphk2-kd cells; ^§§^ *p* < 0.01 compared to the hypoxic Ctrl cells. (**C**) Protein was analyzed by immunoblotting using antibodies against HIF-2α (**C**, top panel), Epo (**C**, middle panel), or β-actin (**C**, bottom panel). Corresponding bands were evaluated by using Image Studio Lite software (version 3.1, LI-COR Biosciences, Lincoln, NE, USA) and normalized to β-actin and are presented in Figure S12.

In a final approach, we aimed to confirm our results in an independent human cell line. Hep3B hepatoma cells are well-established to induce Epo under hypoxic conditions [55]. A stable hSphk2 knockdown was established in Hep3B cells as shown by the 80% reduction of hSphk2 mRNA (Figure 8A). Exposure of control Hep3B cells for 6 h to hypoxia, induced HIF-2α stabilization (Figure 8C, top panel) and enhanced Epo mRNA (Figure 8B) and protein levels (Figure 8C, middle panel). In contrast, in hSphk2-kd cells, HIF-2α and Epo was increased under hypoxia as compared to control cells (Figure 8C). A similar effect was seen on Epo mRNA level (Figure 8B).

## 3. Discussion

In this study, we show for the first time that depletion or catalytic inhibition specifically of Sphk2 upregulated Epo synthesis in renal interstitial fibroblasts.

Sphk2 has previously been attributed a role in the pathogenesis of renal fibrosis. Evidence derived from studies in two mouse models of renal fibrosis, i.e., the UUO model affecting especially the tubular system and causing tubulointerstitial fibrosis, and the streptozotocin-induced diabetic nephropathy model, initially affecting the glomerulus to cause glomerulosclerosis, and later extending to the tubular system to cause tubulointerstitial fibrosis. In both models, *Sphk2−/−* mice showed reduced disease symptoms [12,13,14,15]. The detailed mechanistic basis for this protection is still not completely clear. In the glomerular system, it was shown that loss of Sphk2 enhanced the expression of the podocyte-specific protein nephrin and its regulating transcription factor Wilms tumor suppressor gene 1 (WT1) [15], while in the tubular system, several possible mechanisms were proposed such as an increased production of anti-fibrotic interferon γ by lymphocytes [13], increased protein expression of the anti-fibrotic Smad7 in tubular cells including interstitial fibroblasts [12], or reduced inflammation by promoting macrophage differentiation towards the anti-inflammatory M2 subtype [14].

Since CKD and interstitial fibrosis are often coupled to the development of Epo-deficiency anemia, it is obvious that all intervention strategies that reduce CKD progression will also reduce the development of anemia. Remarkably, our data suggest that loss or inhibition of Sphk2 can on the one side reduce fibrosis [12] and thereby reduce the speed of anemia development, and on the other side can also actively increase Epo synthesis. Whether a decrease in Sphk2 activity also increases Epo in healthy subjects resulting in erythrocytosis, is not yet known. Based on our results in healthy mouse kidneys, *Sphk2−/−* showed increased renal Epo protein expression, increased plasma Epo, increased hemoglobin, and a trend to increased red blood cell numbers. Clearly, more studies are needed to clarify the in vivo relevance of our data.

Our data underline the importance of using Sphk2-selective inhibitors because inhibition of the second enzyme, Sphk1, has an opposite outcome on renal Epo synthesis. Such an opposite function of the two Sphks has also been proposed in other systems. In this view, Sphk1 is mainly reported as an enzyme mediating cell survival and growth, while Sphk2 can have both pro-apoptotic and pro-proliferative effects. Regarding ceramide de novo synthesis, an opposite role of Sphk1 and Sphk2 was reported, as increased expression of Sphk2 led to a higher palmitate incorporation into C16-ceramide whereas Sphk1 rather decreased the incorporation [23]. It has further been proposed that the two Sphks are cross-regulating each other. Thus, the loss of *Sphk*2 led to a compensatory upregulation of Sphk1 in various cell types [20,21,22].

Based on our data, we propose the following mechanism of Epo regulation by Sphk2: inhibition or downregulation of Sphk2 leads to an immediate accumulation of sphingosine, which feeds into the Sphk1 system to produce more iS1P which is exported by the S1P transporter Spns2 or another transporter. Exported S1P will then act as an autocrine ligand of S1P_1_ and S1P_3_ receptors to promote HIF-2α stabilization and increase Epo synthesis, like previously described [44]. This proposed mechanism is underlined by the findings that: (i) the enhanced Epo synthesis in *Sphk2−/−* is abolished by the combined addition of the selective S1P_1_ and S1P_3_ antagonists NIBR-0213 [49] and TY52156 [50]; (ii) the addition of exogenous Sph also enhances HIF-2α and Epo, which is antagonized by NIBR-0213 and TY52156; (iii) blockade of both Sphks prevents iS1P generation and export, and decreases HIF-2α and Epo; and (iv) blockade of both kinases also prevents exogenous Sph-increased HIF-2α and Epo.

A similar way of HIF-1/2 regulation by S1P was previously reported for various cancer cells [56,57]. In these studies, exposure to hypoxia upregulated Sphk1 to produce iS1P, which was exported by Spns2 to allow a subsequent autocrine action via S1P_1_, to stabilize HIF-1/2α and thereby drive a more aggressive cancer phenotype [58]. The same authors also showed that the immunomodulatory drug fingolimod, by mediating a sustained internalization and degradation of S1P_1_, abolished HIF-1α and HIF-2α protein expression in renal carcinoma cells [59]. An additional way of HIF-1/2 regulation by S1P was forwarded by Hait and coworkers [60]. These authors showed an association of Sphk2 with HIF-1α in protein complexes and a direct interaction of S1P with the PAS domain of HIF-1α, which promoted the transcriptional activity of HIF-1α [60]. In that study, downregulation of Sphk2 reduced hypoxia-stimulated HIF-1α stabilization [60]. These authors mainly investigated HIF-1α, but suspected a similar regulation of HIF-2α. Such a mechanism is not consistent with our data, as we show that S1P generated intracellularly by Sphk1 is exported and acts in an autocrine manner through its receptors.

Besides this action of S1P through its receptors, our data also suggest that a specific subcellular pool of Sph can suppress HIF-2α stabilization and Epo synthesis. It is currently unclear by which mechanism this inhibition occurs. However, in view of the well-known function of Sph as a PKC inhibitor [61], it is tempting to speculate that PKC inhibition is a key step in HIF-2α destabilization and reduced Epo synthesis. Supporting this idea, it was reported that in the hepatoma cell line HepG2, PKC has a permissive effect on hypoxia-induced Epo synthesis [62]. It was proposed that the PKC-α isoform is responsible for this action, but other PKC isoforms have also been suggested in other cell types [63,64].

Our data also show that Epo is completely reduced in *Sphk1−/−* cells and that the enhanced Epo by Sphk2 deficiency is attenuated by the Sphk1 inhibitor PF543. This discovery could be significant for certain pathologies where the erythropoietic effect is not desirable, such as secondary congenital erythrocytosis where disease develops due to mutations in genes regulating Epo such as *VHL, EGLN1, EPAS1*, or *EPO* [65]. Further experiments need to be performed to validate whether this treatment strategy poses practical benefits for these groups of patients.

## 4. Materials and Methods

### 4.1. Chemicals

All chemicals are indicated in the Supplementary File.

### 4.2. Cell Lines and Cell Culture Conditions

The mouse REPC-derived cell line FAIK F3-5 was isolated and characterized as previously described [32]. Primary cultures of renal fibroblasts were isolated from C57BL76 mice (Wt), and *Sphk1−/−* and *Sphk2−/−* mice as previously described [66]. *Sphk1−/−* (Sphk1^tm1geno^) and *Sphk2−/−* (Sphk2^tm1geno^) mice were generated by GenOway S.A. (Lyon, France). Both cells types were cultured in Dulbecco’s modified Eagle’s medium (DMEM) supplemented with 10% (*v*/*v*) fetal bovine serum (FBS), 10 mM HEPES pH 7.4, 100 units/mL penicillin and 100 µg/mL streptomycin.

The human hepatoma cell line Hep3B was obtained from the European Collection of Authenticated Cell Cultures (ECACC) through Sigma Aldrich. Hep3B cells were cultured in DMEM supplemented with 10% (*v*/*v*) fetal bovine serum (FBS), 10 mM HEPES pH 7.4, 100 units/mL penicillin and 100 µg/mL streptomycin. All cells were grown at 37 °C in a humidified atmosphere containing 5% CO_2_. Prior to stimulation, cells were rendered serum-free in DMEM containing 10 mM HEPES and 0.1 mg/mL fatty acid-free bovine serum albumin (BSA) for 16 or 24 h unless otherwise stated.

### 4.3. Stable mRNA Knockdown in F3-5 and Hep3B Cells

Stable knockdown of mSphk1 and Sphk2 mRNA in F3-5 cells was generated by transducing cells with 5 or 4 different shRNA constructs according to the manufacturer’s instructions (Sigma MISSION^®^ (Merck KGaA, Darmstadt, Germany); mSphk1: TRCN0000024684 (sh1), TRCN0000024685 (sh2), TRCN0000024686 (sh3), TRCN0000024687 (sh4), and TRCN0000024688 (sh5); mSphk2: TRCN0000024629 (sh1), TRCN0000024631 (sh3), TRCN0000024632 (sh4), and TRCN0000024633 (sh5)). Transduction was performed in the presence of 6 μg/mL polybrene. Stable clones were selected by 1 µg/mL puromycin. Best knockdown for mSphk1 was achieved with sh5, further denoted as mSphk1-kd. Best knockdown for mSphk2 was achieved with a combination of sh1, sh3, and sh4. This cell line was further denoted mSphk2-kd. Stable knockdown of hSphk2 in Hep3B cells was achieved by transduction of cells with a lentiviral shRNA expression construct against hSphk2 (Sigma MISSION^®^; TRCN0000036970 in a pLKO.1-puro vector).

### 4.4. Cell Stimulation, Homogenization, and Immunoblotting

For hypoxia exposures, a hypoxia chamber (Whitley H35 HEPA Hypoxystation; Don Whitley Scientific) was used at 1% oxygen and 5% CO_2_. Cell stimulation and homogenization was done exactly as previously described [44]. Protein lysates containing equal amounts of protein were separated by SDS-PAGE, transferred to nitrocellulose membranes by wet blotting using a buffer containing 25 mM Tris-HCl pH 8.3, 190 mM glycine and 20% (*v*/*v*) methanol, or by semi-dry Fast blotting (Bio-Rad Laboratories Inc., Hercules, CA, USA) with the recommended buffer. Membranes were blocked with 3% (*w*/*v*) low-fat milk powder in PBS for 1 h and were then incubated for 18–24 h at 4 °C with the respective antibodies and diluted in a buffer containing 50 mM Tris-HCl pH 7.4, 200 mM NaCl, 10% (*v*/*v*) horse serum, 3% (*w*/*v*) BSA fraction V, and 0.1% (*v*/*v*) Tween20. Secondary fluorescent antibodies were from LI-COR Biosciences (Lincoln, NE, USA), and development was done in a Licor fluorescence-chemiluminescence detector. Bands were evaluated by using the Image Studio Lite software (LI-COR Biosciences, Lincoln, NE, USA) or the ImageJ software (National Institutes of Health, MA, USA). Antibodies derived against the following proteins were used: Epo (St. John’s Laboratory, London, UK, STJ27630, 1:1000), HIF-2α (Bethyl Laboratories A700-003, 1:1000), β-actin (Sigma Aldrich/Merck KGaA, Dramstadt, Germany, A-1978, 1:5000), α-tubulin (Sigma Aldrich/Merck KGaA, Darmstadt, Germany, T9026 1:3000), α-smooth muscle actin/ACTA2 (Proteintech, Rosemont, IL, USA, 14395-1-AP, 1:1000); IRDye 800CW secondary antibodies were from LI-COR Biosciences (Lincoln, NE, USA). Antibodies against mSphk1 and mSphk2 were generated as previously described [22].

### 4.5. RNA Extraction and Quantitative PCR Analysis

RNA extraction was done exactly as previously described [44]. First strand cDNA was synthesized using 2 µg of total RNA as template for reverse transcription (RT). SYBRGreen-based quantitative PCR (qPCR) was performed in a BioRad CFX Connect™ Optics Module thermal cycler (Bio-Rad Laboratories Inc., Hercules, CA, USA). The Bio-Rad CFX Manager software (version 3.1., Bio-Rad Laboratories Inc., Hercules, CA, USA) was used to monitor the melting curve, and to obtain the quantification data. The relative mRNA expression of the gene of interest was calculated with the ∆∆Ct method normalized to ribosomal protein L28 mRNA or 18S rRNA as housekeeping genes. The following primers were used: mEpo: forward: AAT GGA GGT GGA AGA ACA GG, reverse: ACC CGA AGC AGT GAA GTG A; mL28 forward: GCA AAG GGG TCG TGG TAG TT, reverse: TTC TGG CTT CGA AGG ATG GC; mSphk1: forward: ACA CTG CTT CTG GGC TGC, reverse: GTT ATG GTT CTT CTG GAG GTG G; mSphk2: forward: GCA CGG CGA GTT TGG TTC C, reverse: TGT ATG TGT AGG GCT TGT GTT GTG; hSphk2: forward, TCA ACC TCA TCC AGA CAG AAC GAC, reverse: CAT CCC ACT CAC TCA GGC TCA G; hEpo: forward: TGG GGG TGC ACG AAT GT, reverse: TTT GGT GTC TGG GAC AGT GA; hL28: forward: GCA ATT CCT TCC GCT ACA AC, reverse: TGT TCT TGC GGA TCA TGT GT; 18S: forward: CGA TTC CGT GGG TGG TGG TG, reverse: CAT GCC AGA GTC TCG TTC GTT ATC.

### 4.6. Sphingolipid Quantification by Mass Spectrometry

Confluent cells in 60-mm-diameter dishes were scraped in methanol and taken for lipid extraction and quantification of the different sphingolipids as previously described [48].

### 4.7. Red Blood Cell Counting

All animal experiments were approved by the committee of animal experimentation of the Veterinary Department of the Canton of Bern, under approval number BE-50/17. C57BL/6J mice were provided by Janvier Labs (Le Genest-Saint-Isle, France). Sphk2^tm1geno^ (*Sphk2−/−*) were generated by GenOway S.A. (Lyon, France). Blood was collected in EDTA-K3-coated microvettes (Sarstedt, Sevelen, Switzerland) from the tail vein of 8-week old C57BL/6J (Wt) or *Sphk2−/−* mice and the number of RBC and hemoglobin were assessed with a Scil Vet ABC^TM^ Hemocytometer (Scil Animal Care Company GmbH; Viernheim, Germany).

### 4.8. Statistical Analysis

Statistical analysis was performed by one-way ANOVA or an unpaired *t*-test, where applicable. For multiple comparisons, a Bonferroni post hoc test was used. GraphPad Prism Software (version 8.4.3., Prism-GraphPad, San Diego, CA, USA) was used for graph presentations and statistical analysis.

## 5. Conclusions

In summary, our study has shown that Sphk2 is a putative novel pharmacological target in the renal Epo synthesis path, suggesting that selective inhibition of Sphk2 may not only reduce the progression of fibrosis in CKD, but may also directly increase Epo synthesis and prevent the development of anemia in CKD patients.

## Data Availability

The authors declare that all data supporting the findings of this study are available within this paper or within the Supplementary File or can be obtained from the corresponding author up on request.

## Supplementary Materials

The following supporting information can be downloaded at: https://www.mdpi.com/article/10.3390/ijms23115882/s1.

## Abbreviations

α-SMA	α-smooth muscle actin
CKD	chronic kidney disease
CTGF	connective tissue growth factor
DMEM	Dulbecco’s modified Eagle medium
Epo	erythropoietin
FBS	fetal bovine serum
GPCR	G protein-coupled receptor
HIF	hypoxia-inducible factor
kd	knockdown
MAPK	mitogen-activated protein kinase
PBS	phosphate-buffered saline
PKC	protein kinase C
qPCR	quantitative polymerase chain reaction
REPC	renal Epo-producing cells
S1P	sphingosine 1-phosphate
SDS-PAGE	sodium dodecyl sulfate polyacrylamide gel electrophoresis
shRNA	small hairpin RNA
Sph	sphingosine
SphK	sphingosine kinase
UUO	unilateral ureteral obstruction
