# Supplementary material for: Sphk1 and Sphk2 Differentially Regulate Erythropoietin Synthesis in Mouse Renal Interstitial Fibroblast-like Cells
Source: Int J Mol Sci. 2022 May 24;23(11):5882. doi: 10.3390/ijms23115882 (PMC9180811; doi:10.3390/ijms23115882)
Supplement: Supplementary file 1 [file ijms-23-05882-s001.zip › ijms-1643852-supplementary.pdf]

Supplementary data file:

Hafizi et al.
Sphk1 and Sphk2 differentially regulate erythropoietin synthesis in mouse renal interstitial fibroblast-like cells.

Chemicals and reagents:

PF543 and CGP41251 (PKC412) were purchased from Selleckchem (Houston, TX, USA); SLM6031434/HCl and TY52156 were from Tocris Bioscience (Bristol, UK); HWG-35D was kindly provided by Prof. Nigel Pyne (Glasgow, U.K.); sphingosine was from Cayman Chemicals/Adipogen AG (Liestal, Switzerland); NIBR-0213 was from GLixx Laboratories (Hopkinton, MA, USA); RO-318220 was from Labforce AG (Muttenz, Switzerland); the mouse Epo ELISA was from Biolegend/Lucerna-Chem AG (Luzern, Switzerland); Kapa SYBR FAST, fatty acid-free bovine serum albumin (BSA), BSA fraction V, deferoxamine, puromycin, dimethyl sulfoxide, and horse serum were from Sigma-Aldrich Chemie GmbH (Buchs, Switzerland). RNA-Solv® reagent from Omega Bio-tek Inc., (Norcross, GA, USA); First Strand cDNA Synthesis kit was from Thermo Scientific (Waltham, MA, USA). IRDye® 800CW secondary antibodies were from LI-COR Biosciences (Lincoln, NE, USA). Trypsin-EDTA 0.25%, DMEM containing Glutamax™, pyruvate and 4.5 g/L D-glucose were from Gibco® by Life Technologies Limited (Paisley, UK). Fetal bovine serum (FBS) was purchased from PANBiotech GmbH (Catalogue No. P40-37, Aidenbach, Germany). Transblot Turbo RTA transfer kit was from BioRad Laboratories (Hercules, CA, USA). All oligonucleotide primers were from Eurofins Genomics GmbH (Ebersberg, Germany). Standard chemicals were of highest possible grade and either from Carl Roth GmbH (Karlsruhe, Germany) or from Sigma-Aldrich Chemie GmbH (Buchs, Switzerland).

Supplementary data:

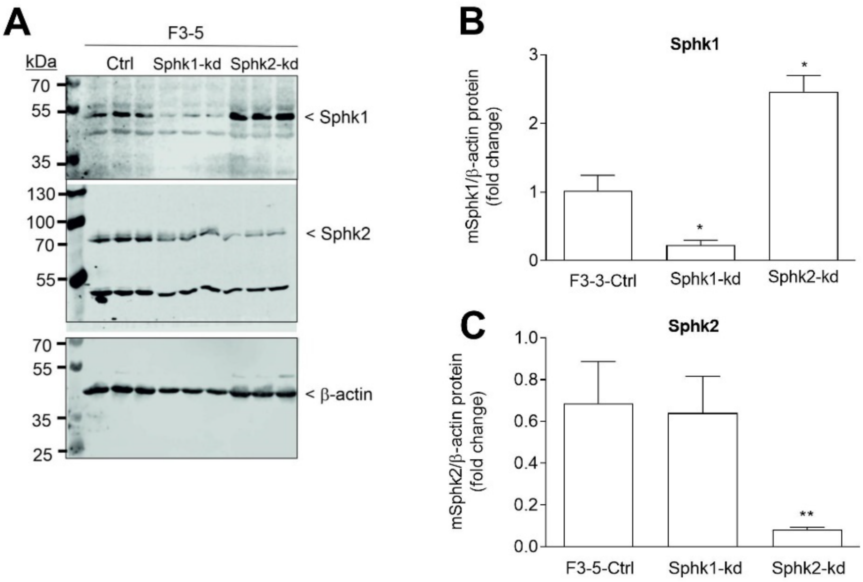

Figure S1

F3-5 fibroblasts, that were stably transduced with an empty vector (Ctrl), or a vector containing shRNA specific for Sphk1 (Sphk1-kd) or Sphk2 (Sphk2-kd), were incubated for 16 h in serum-free DMEM. Protein extracts were prepared and analysed by immunoblotting using antibodies against mouse Sphk1 (A, upper panel), mouse Sphk2 (A, middle panel), or β-actin (A, bottom panel). Corresponding bands were evaluated by using Image Studio Lite software, normalized to β-actin, and are presented in B (for Sphk1) and C (for Sphk2). Results are expressed as fold change compared to Ctrl and are means + S.D. (n=3). *p < 0.05, **p < 0.01, ***p < 0.001 were considered statistically significant when compared to the Ctrl samples.

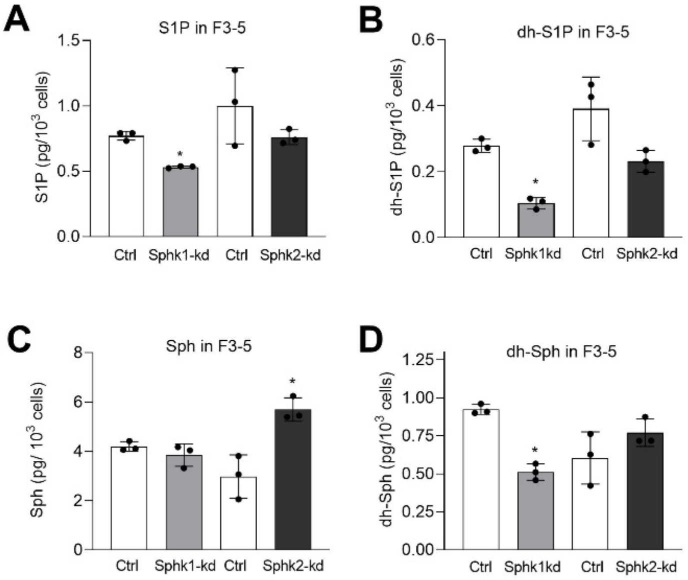

Figure S2

Confluent cells in 60 mm-diameter dishes were incubated for 16 h with serum-free DMEM and then taken for lipid extraction and LC/MS analysis of S1P (A), dihydro (dh)-S1P (B), Sph (C) and dh-Sph (D). Results are indicated as pg lipid/ 10^3 cells and are means ± S.D. (n=3). *p < 0.05 was considered statistically significant when compared to the respective Ctrl samples.

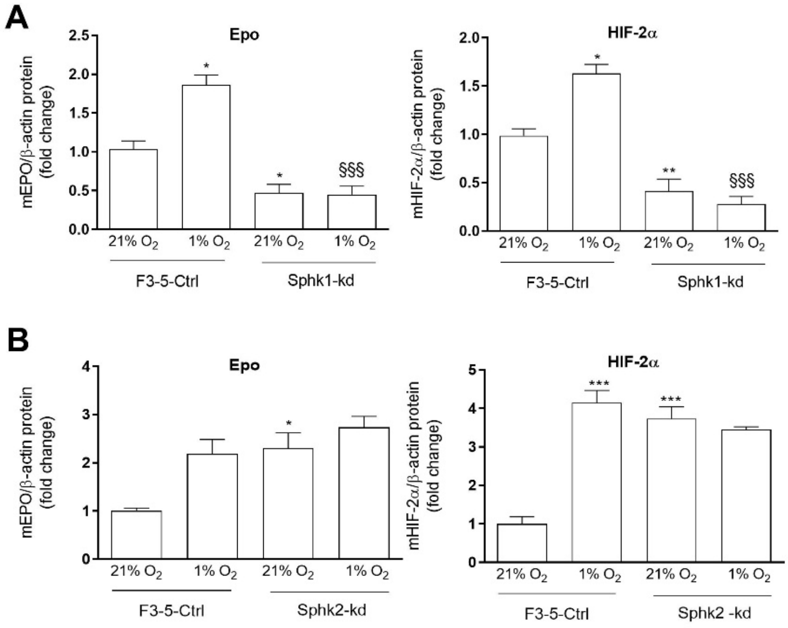

Figure S3

Blots in Figure 2 were evaluated by Image Studio Lite software. Bands corresponding to 119 kDa HIF-2α and 34 kDa Epo protein were normalized to β-actin. A shows the F3-5-Ctrl versus Sphk1-kd cells; B shows the F3-5-Ctrl versus Sphk2-kd cells. Results are expressed as fold change compared to normoxic controls and are means + S.D. (n=3). *$p < 0.05$, ***$p < 0.001$ were considered statistically significant when compared to the normoxia Ctrl samples; §§§$p< 0.001$ compared to the hypoxic Ctrl samples.

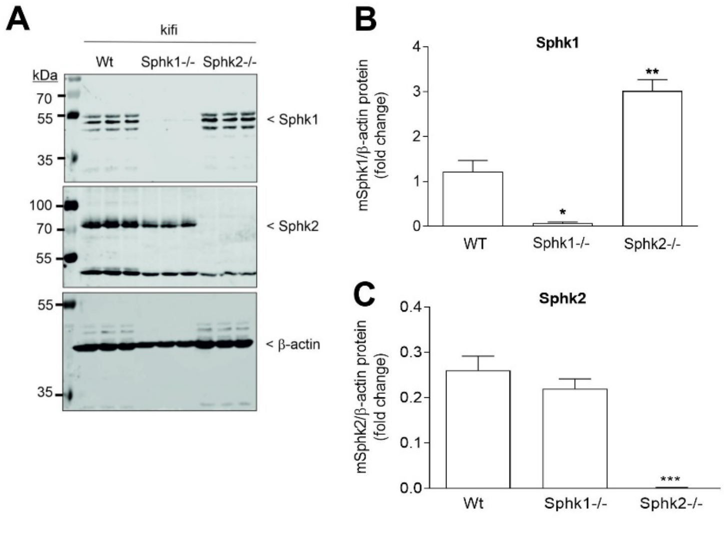

Figure S4

Primary cultures of renal fibroblasts (kifi) isolated from either C57BL/6 (Wt), *Sphk1-/-* or *Sphk2-/-* mice were incubated for 16 h in serum-free DMEM. Protein extracts were prepared and analysed by immunoblotting using antibodies against mouse Sphk1 (A, upper panel), mouse Sphk2 (A, middle panel), or β-actin (A, bottom panel). Corresponding bands were evaluated by using Image Studio Lite software, normalized to β-actin, and are presented in B (for Sphk1) and C (for Sphk2). Results are expressed as fold change compared to WT and are means + S.D. (n=3). *$p < 0.05$, **$p < 0.01$, ***$p < 0.001$ were considered statistically significant when compared to the WT samples.

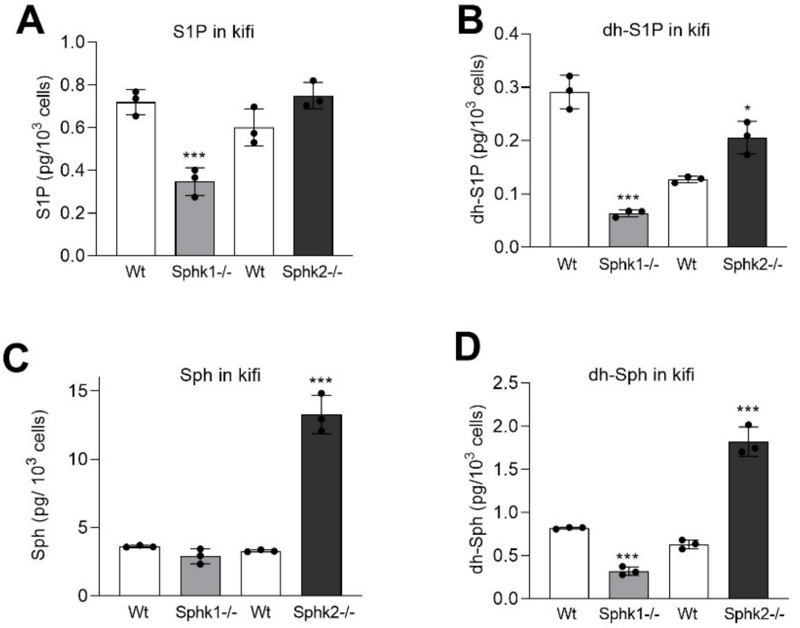

Figure S5

Confluent cells in 60 mm-diameter dishes were incubated for 16 h with serum-free DMEM and then taken for lipid extraction and LC/MS analysis of S1P (A), dihydro-S1P (B), Sph (C) and dihydro-Sph (D). Results are indicated as pg lipid/ 10^3 cells and are means ± S.D. (n=3). *p < 0.05, ***p < 0.001 were considered statistically significant when compared to the respective Wt samples.

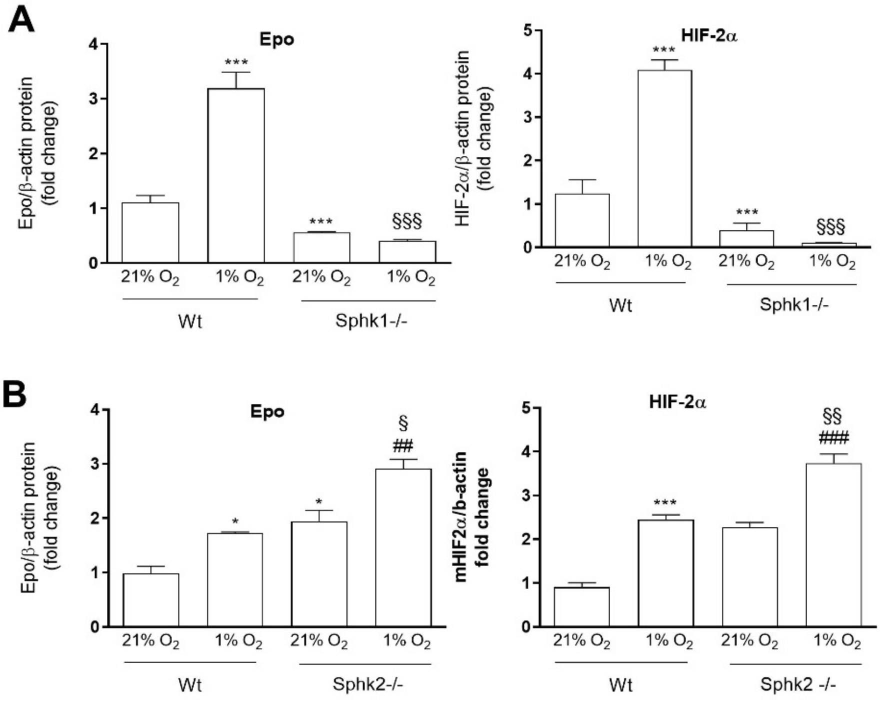

Figure S6

Blots in Figure 4 were evaluated by Image Studio Lite software. Bands corresponding to 119 kDa HIF-2α and 34 kDa Epo protein were normalized to β-actin. A shows the Wt versus *Sphk1-/-* cells; B shows the Wt versus *Sphk2-/-* cells. Results are expressed as fold change compared to controls and are means + S.D. (n=3). *p< 0.05, ***p< 0.0001 were considered statistically significant when compared to the normoxia Wt samples; ##p< 0.01, ###p< 0.001 when compared to the normoxia -/- samples; §p< 0.05, §§p< 0.01 §§§p< 0.001 when compared to the hypoxia Wt samples.

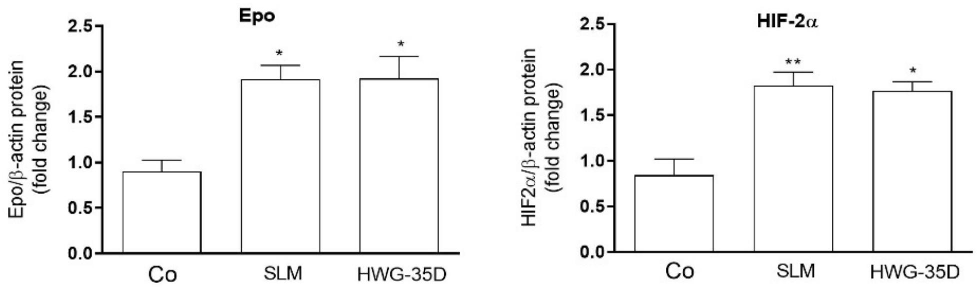

Figure S7

Blots in Figure 5 were evaluated by Image Studio Lite software. Bands corresponding to 119 kDa HIF-2α and 34 kDa Epo protein were normalized to β-actin. Results are expressed as fold change compared to controls and are means + S.D. (n=3). *p< 0.05, **p< 0.01 were considered statistically significant when compared to the Co samples.

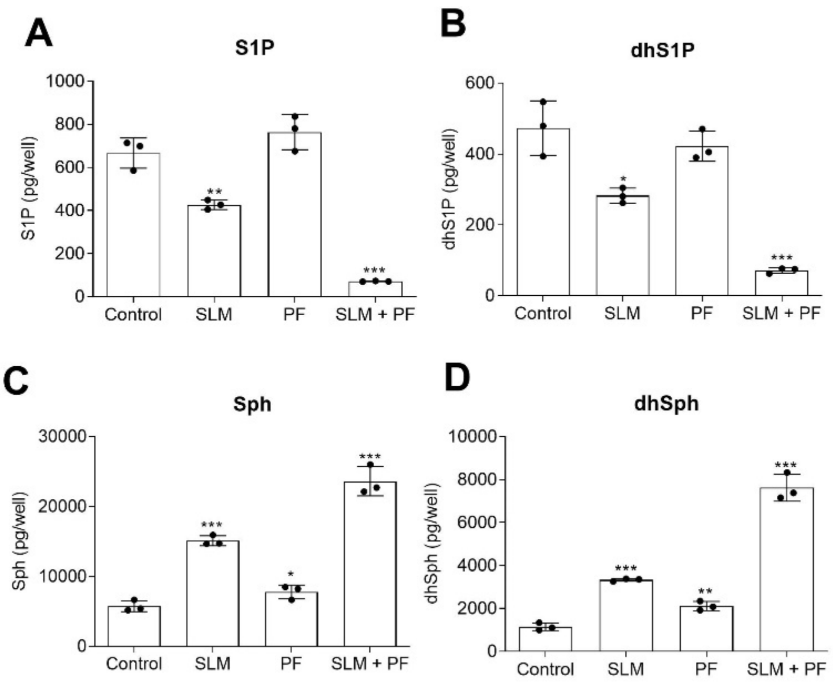

Figure S8

Confluent cells in 60 mm-diameter dishes were stimulated for 6 h with vehicle (control), SLM6031434 (SLM, 3 μM), PF543 (PF, 1 μM), or SLM plus PF543 (3 μM+1 μM). Cell monolayers were taken for lipid extraction and LC/MS analysis of S1P (A), dihydro-S1P (B), Sph (C) and dihydro-Sph (D). Results are indicated as pg lipid/ well and are means ± S.D. (n=3). *p < 0.05, **p<0.01, ***p<0.001 were considered statistically significant when compared to the control samples.

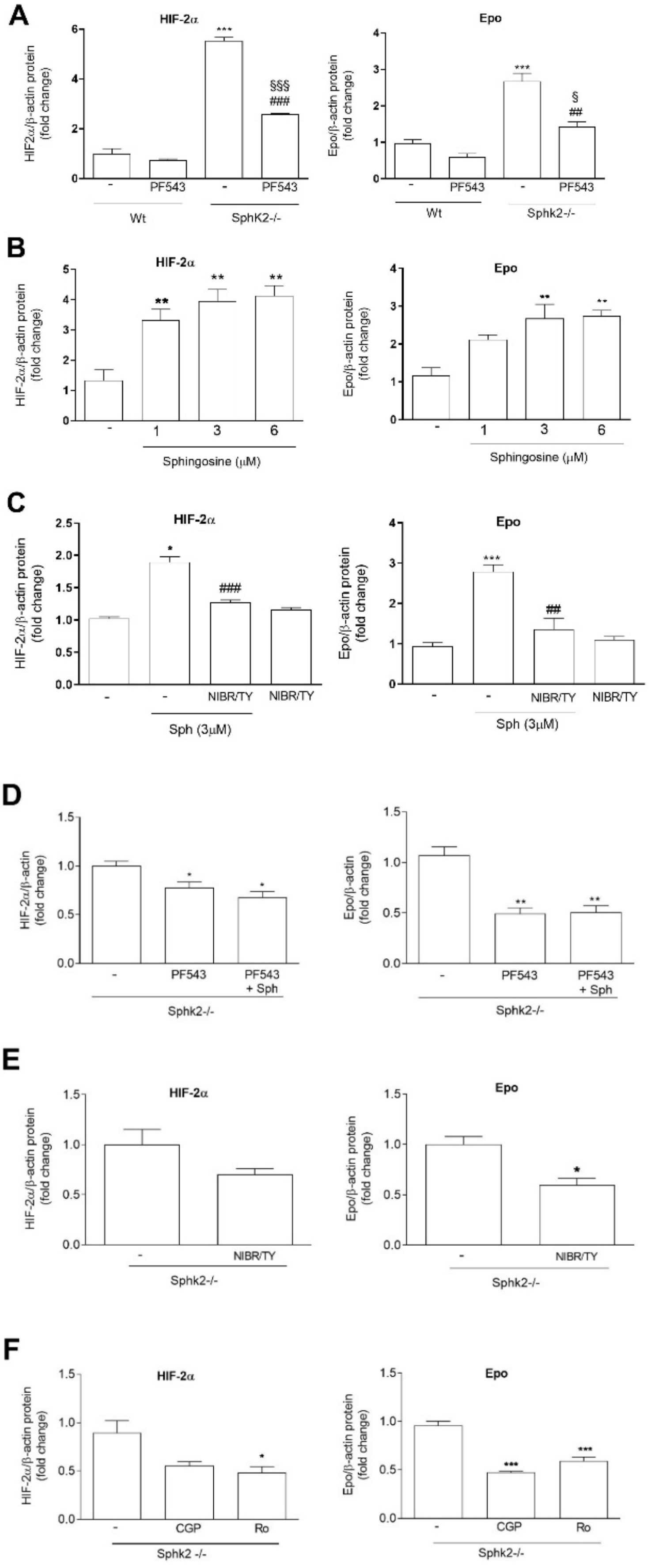

Suppl. Figure S9

Blots in Figure 6 were evaluated by Image Studio Lite software. Bands corresponding to 119 kDa HIF-2α and 34 kDa Epo protein were normalized to β-actin. Results are expressed as fold change compared to controls and are means + S.D. (n=3). *$p < 0.05$, **$p < 0.01$, ***$p < 0.001$ were considered statistically significant when compared to the control samples; #$p < 0.05$, ##$p < 0.01$, ###$p < 0.001$ when compared to the *Sphk2-/-* samples (in A), to the Sph-stimulated samples (in C), to the untreated samples (in D); §$p < 0.05$, §§§$p < 0.001$ when compared to the PF543-treated Wt samples (in A).

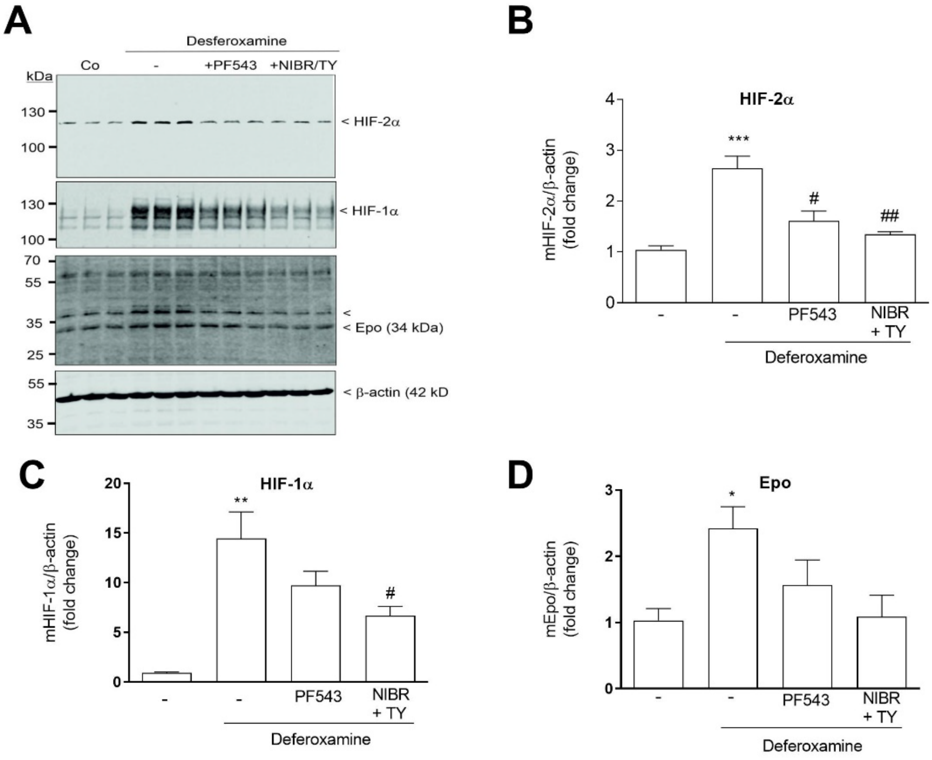

Figure S10

Wildtype mouse renal fibroblasts were treated for 24 h with deferoxamine (100 µM) in the absence (-) or presence of PF543 (1 µM) or NIBR-0213 plus TY52156 (10 µM each). Proteins were analysed by immunoblotting using antibodies against HIF-2α (A, top panel), HIF-1α (A, middle panel), Epo (middle panel), and β-actin (bottom panel). Corresponding bands were evaluated by using Image Studio Lite software, normalized to β-actin. Results are expressed as fold change compared to controls and are means + S.D. (n=3). **$p< 0.01$, *** $p< 0.001$ were considered statistically significant when compared to the control values; #$p< 0.05$ ##$p< 0.01$ compared to the deferoxamine-treated values.

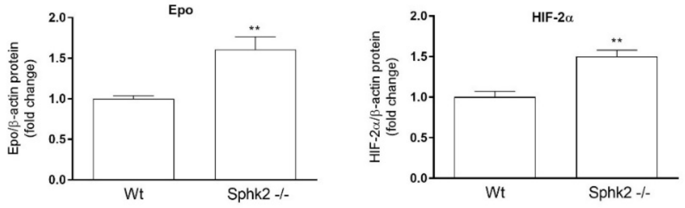

Figure S11

Blots in Figure 7 were evaluated by Image Studio Lite software. Bands corresponding to 119 kDa HIF-2α and 34 kDa Epo protein were normalized to β-actin. Results are expressed as fold change compared to controls and are means + S.D. (n=3). **$p< 0.01$ were considered statistically significant when compared to the Wt samples.

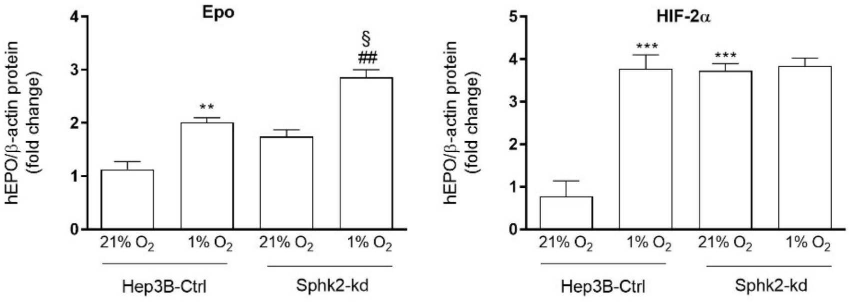

Figure S12

Blots in Figure 8 were evaluated by Image Studio Lite software. Bands corresponding to 119 kDa HIF-2α and 34 kDa Epo protein were normalized to β-actin. Results are expressed as fold change compared to controls and are means + S.D. (n=3). **p< 0.01, ***p< 0.001 were considered statistically significant when compared to the normoxia Ctrl samples; ##p< 0.01 when compared to the normoxia kd samples; §p< 0.05 when compared to the hypoxia Ctrl samples.
